# Study of the Knowledge of Pediatricians and Senior Residents Relating to the Importance of Hearing Impairment and Deafness Screening Among Newborns 

**Published:** 2014-04

**Authors:** Mehrdad Rogha, Elham Mokhtari

**Affiliations:** 1 *Department of Otorhinolaryngology, School of Medicine, Isfahan University of Medical Sciences, Isfahan, Iran.*

**Keywords:** Hearing impairment, Hearing screening, Early detection, Intervention

## Abstract

**Introduction:**

Newborn hearing screening leads to the early detection of hearing impairment. The aim of screening is to decrease or remove the effect of hearing impairment on development of speech and language by timely diagnosis and effective treatment. A number of risk factors lead to delayed start of decreased hearing ability including: 1. Congenital infection with cytomegalovirus (CMV) virus, 2. Meningitis, 3. Mumps, 4. Positive family history, 5. Head trauma, 6. Chemotherapy,7. Syndrome pertaining to delayed start of decreased hearing. Unfortunately, lack of attention to early diagnosis of hearing impairment is becoming a general health problem. No research has yet been carried out relating to the knowledge of pediatricians on this issue, particularly the importance of hearing impairment and hearing screening. The aim of this study was to determine the attitude to newborn hearing screening among pediatricians.

**Materials and Methods:**

This cross-sectional, descriptive-analytic study was conducted in Isfahan in 2012 among 300 pediatricians and final-year pediatric residents. An adjusted 22-question version of the Early Hearing Detection and Intervention (EHDI) questionnaire was used to collect data. The validity and reliability of the EHDI questionnaire was previously demonstrated by Boys Town National Research Hospital and its Farsi translated version was validated by the EDC Center at the Isfahan University of Medical Sciences.

**Results:**

In our study, 83% of pediatricians agreed on the importance of hearing impairment screening for all infants. However 65% were not aware of special needs for hearing-impaired patients.

**Conclusion:**

Newborn hearing impairment and deafness screening is important, irrespective of the costs, and lack of timely diagnosis results in both individual and social consequences. The majority of physicians use textbooks to gain information about hearing screening, but recognize that this is insufficient. Although it is now one of the most useful tools for gathering and applying new information, the physicians in our study rely very little on the Internet as a source of information.

## Introduction

Hearing impairment, a reduction in hearing ability, tends to be an invisible and hidden human disability, but its consequences can be very severe (1,2). It is also one of the most prevalent congenital disorders among infants (3), and unfortunately it is not detected easily by ordinary clinical methods (4). Nearly half of all infants suffering from hearing impairment have none of the known risk factors (5), while US data show that nearly 95% of the parents of hearing-impaired children suffer no hearing disability themselves (6).

Lack of timely diagnosis of hearing impairment among infants can lead to considerable individual and social complications including (7,8):

1. Delay in natural development of speech and language and speech disorders.

2. Learning disorders, academic failure, reading and writing problems.

3.Communicational and emotional-behavioral disorders, delay in social-cultural growth.

4. Unsuitable communication with parents and friends, misunderstanding and bad behavior towards these people.

A Joint Committee of the American Academy of Pediatricians has recommended that all infants be screened by hearing tests to diagnose of hearing impairment early (9). Timely detection of hearing impairment among children followed by timely therapeutic intervention improves levels of health, as well as the child's potential in all fields of growth and cognitive ability (10).

As primary care physicians, general practitioners (GPs) are the main route to educate and encourage parents to seek hearing screening and also to follow-up hearing-impaired infants. This is because GPs understand the concept of hearing screening the importance hearing and early detection of hearing disorders, and appropriate routes for patient referral (11). Olusanya et al. conducted an opinion poll among US physicians on the subject of hearing screening and concluded that the principal challenges were limited time for visits, insufficient knowledge, and need for educational courses (12).

The principal motivation in newborn hearing screening programs is early detection of hearing impairment and accordingly early intervention for rehabilitation. Yoshinaga-Itano et al. reported that infants diagnosed before 6 months who participated in rehabilitation programs have better acquired skills such as pronunciation, understanding, mathematical reading, influential social behaviors, and communication with people other than their families (13,14).

Lack of hearing impairment diagnosis and late therapeutic intervention is often accompanied by increasing learning risks, greater treatment costs, and loss of productivity (10,11,13).

Since pediatricians are at the frontline in dealing with hearing-impaired children and their families, the current research aims to study the knowledge of specialists and final-year residents in terms of the importance of hearing impairment and deafness screening.

## Materials and Methods

This cross-sectional, descriptive-analytic study was conducted in 2012 in Isfahan. The target population was all pediatricians and final-year pediatric residents responsible for the care of infants. According to statistical reports of the Isfahan Vice Chancellor for education and medical treatment, approximately 290 qualified pediatricians and 10 senior assistants in pediatrics were working in this city at the time the study was conducted.

Therefore, the inclusion criteria were pediatricians and final-year pediatrics assistants practicing in infant care, who declared their consent to participate in the study. An adjusted 22-question version of Early Hearing Detection and Intervention (EHDI) questionnaire was used to collect data. Validity and reliability of EHDI questionnaire have previously been demonstrated by Boys Town National Research Hospital and its Farsi translated version was validated by the EDC Center at the Isfahan University of Medical Sciences (9).

Questionnaires in addition to required descriptions were given to pediatricians at their workplace, and completed forms were returned during a subsequent visit. Collected data were analyzed by SPSS20. As there was no drug intervention in this study, this research is consistent with medical ethical issues. Close to twenty-five pediatricians did not participate in this study because of a lack of time In response to the question, “What percentage of your practice is accounted for by infants?”, 17 (5.7%), 74 (24.6%), 92 (30.7%), and 117 (39%) pediatricians responded >75%, 50–75%, 25–50%, and <25% of respectively. In response to the question, “How important is screening of infants for permanent hearing impairment”, 25 (83%) considered it important, 38 (12.7%) considered it to be of little importance, and 11 individuals (3.6%) responded,“Unimportant”.

## Results

The mean age of the respondents was 48.2±7.4 years, and the population consisted of 257 (85.7%) males and 43 (14.3%) females. Demographic data for the study population are presented in (Table 1).

**Table 1:** Demographic information of studied physicians

**Age (Mean± SD)**	**7.4** **±48.2 year**	
Sample Groups	Specialist (%)	280(91)
	Resident (%)	20(9)
Gender	Male (%)	257(85.7)
	Female (%)	43(14.3)
Place of Practice	Private office (%)	189(64)
	Hospital (%)	111(37)
Working History	More than 15 years (%)	160(54)
	Between 10 to 15 years (%)	110(36)
	Less than 10 years (%)	30(10)

Results showed that 210 (70%) pediatric- cians had seen between two and five infants with permanent hearing impairment and 90 (30%) had seen fewer that two infants within the previous three years. Further, 291 (98%), 280 (92%), and 5 (2%) doctors had referred infants with permanent hearing impairment to an ear, nose and throat (ENT) specialist, audiologist, and neurologist, respectively.

A total of 285 pediatricians (88%) received more than 90% of referred infants hearing screening results while 15 pediatricians (12%) received no results.

**Fig 1:**
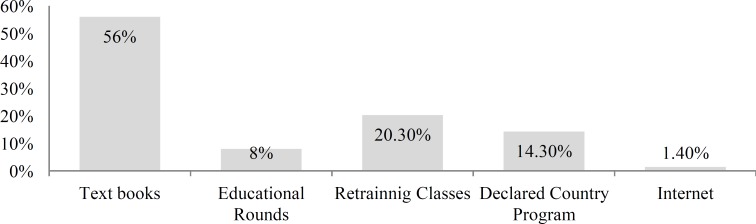
Relative frequency of primary references for hearing screening

**Fig 2:**
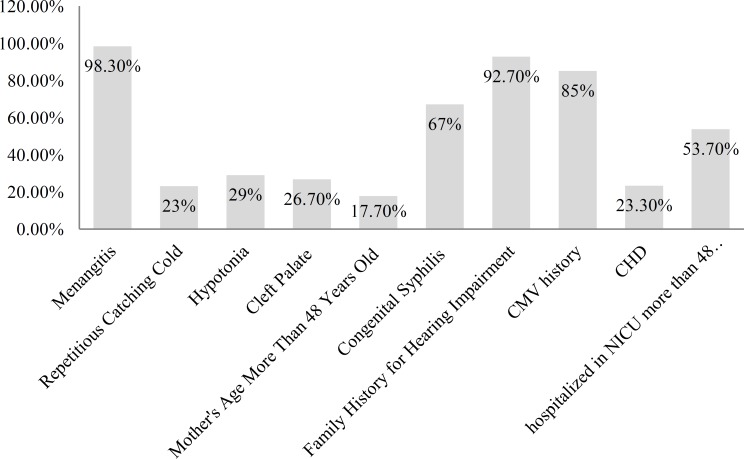
Relative frequency of risk factors for delayed hearing impairment, as reported by the study population

Responses to the question “What are your primary references for hearing screening?” are shown in Figure 1, while Figure 2 shows frequencies of different conditions in response to the question, "Which conditions put the children at risk of delayed hearing impairment? Responses to all other questions are tabulated in (Table 2). 

**Table 2:** Demographic Information of Studied Physicians

**Question**	**Response (%)**	**N (%)**
What percentage of your practices was composed of infants?	Less than 25%	117 (39)
	Between 25% and 50%	92 (30.7)
	Between 50% and 75%	74 (24.6)
	More than 75%	17 (5.7)
		
Infants screening for permanent hearing impairment	Important	251 (83.7)
	of little importance	38 (12.7)
	unimportant	11 (3.6)
		
Dose hearing screening make parents worried?	Yes	27 (9)
	no	248 (82.6)
	I do not know	25 (8.4)
		
How much do you rely on your true description to parents about result of hearing screening?	a great deal	145 (48.3)
	Much	130 (43.3)
	somewhat	25 (8.4)
		
Are you aware of especial needs of hearing-impaired children?	Yes	42 (14)
	Somewhat	63 (21)
	no	195 (65)
		
How much do you use Internet to get information about hearing screening?	A lot	34 (11.3)
	Moderate	77 (25.7)
	A little	189 (63)
		
Is program of early detection and treatment of hearing impairment conducted in our country?	No	25 (8.4)
	Yes	210 (70)
	I do not know	65 (21.6)
		
Is general newborn hearing screening valuable regardless of its cost?	yes	289 (96.4)
	no	11 (3.6)
		
Which infants have you introduced for hearing screening last year?	nearly all infants	65 (21.6)
	only cases with risk factors	235 (78.4)
		
Which conditions make infants candidate for Cochlear Implant?	two-sided mild-to-moderate decreased hearing	46 (15.3)
	two-sided sever decreased hearing	225 (75)
	one-sided sever decreased hearing	12 (4.1)
		17 (5.6)

## Discussion

Hearing impairment is one of the most prevalent congenital disorders and, if it is not detected, it can have an adverse effect on speech, language, and cognition. According to studies in Iranian infants in Tehran published in 2004, it was estimated that 1–4 infants out of 1,000 births in a normal care unit and 2.5–4.5 infants out of 100 births in an intensive care unit have two-sided severe sensorineural hearing impairment (16). Also a study into the frequency of infant hearing impairment in 2008 showed the benefit of an infant hearing screening program in early detection of hearing impairment (17). According to the results of our study, 83.7% of pediatricians considered the screening of all permanently hearing-impaired infants important, and also they accepted that early therapeutic intervention can have a significant effect on improving health status in all developmental and cognitive abilities.

In the study by Nicole et al. investigating physicians in Minnesota in 2006, 80% considered the hearing screening of all infants important and 75% considered screening valuable, regardless of cost. However in our study, 83.7% of pediatricians recognized that hearing screening of all infants is important and 96.4% of them responded yes to the question “Is hearing screening valuable with any expenditure?”. Furthermore, 55% of Minnesotan physicians were sure about their description of the results of screening and sufficient knowledge for dealing with parents. In our study 48.3% were very sure about screening, 43.3% considered they had a lot of knowledge and 8.4% had a moderate knowledge of screening. 

Our results also showed that the most prevalent risk factors for delayed hearing impairment among infants are meningitis (98.3%), family history of hearing impairment (92.7%), history of cytomegalovirus (CMV) infection (85%), congenital syphilis (67%), hospitalization in a neonatal intensive care unit (NICU) for more than 48 hours (53.7%), hypotonia (29%), cleft palate (26.7%), chronic heart disease (23.3%), repeated exposure to cold (23%), and mother's age >48 years old (17.7%). In other studies, the first risk factor was meningitis and then family history of hearing impairment and CMV history. In a study of American physicians reported in 2006 by Moeller et al., 99% of respondents said that the most prevalent risk factors were meningitis, family history of hearing impairment, and CMV history. Moellor also reported that 75.8% of physicians referred permanent hearing-impaired infants to an ENT specialist, 41.3% to an audiologist, 8.9% to a geneticist, and 7.1% to a neurologist. 

However in our study, 98% of physicians referred patients to an ENT specialist, 92% to an audiologist and 2% to a neurologist. Accordingly, we can conclude that our physicians did not refer infants with permanent hearing impairment to a geneticist or do not have sufficient knowledge of this option.

Hearing screening should ideally be performed before the infant reaches the age of 1 month. Detection and use of hearing aid equipment should be carried out before 3 months of age, and therapeutic intervention before the age of 6 months (13,18). This guidance is confirmed by most studies, but in our study fewer than half of the physicians were aware of it and 65% of respondents were not aware of the special needs of hearing-impaired children. Severe hearing impairment has an adverse effect on speech and language skills, but mild-to-moderate hearing impairment or one-sided disease has fewer disadvantages. According to reports of American physicians in 2006, children with one-sided hearing impairment can function independently with a hearing aid, and do not require invasive treatment (8). In our study, 75% of physicians prescribed cochlear implant or other invasive treatment for infants with two-sided severe impaired hearing. 

## Conclusion

Newborn hearing screening programs help to determine the level of decreased hearing ability and allow physicians to take the appropriate action in cooperation with an ENT specialist, pediatrician, neurologist, geneticist, and audiologist. According to the results of this study, physicians understood the importance of infant hearing screening and considered it valuable irrespective of its cost; they were also aware of its role in the prevention of individual and social complications. Therefore, suitable frame- works need to be established to allow infant hearing screening to become a financial possibility. 

According to the results presented herein, the majority of physicians used textbooks to access information relating to hearing screening, but they recognized that this was an insufficient resource. Although it is now one of the most useful tools for gathering and applying new information, the physicians in our study rely very little on the Internet as a source of information. Furthermore, the physicians in our study did not have sufficient knowledge about the special needs of children with hearing impairment, and believed that they could increase their knowledge by using additional sources of reference. 
